# Models of Care and Interventions to Improve Person‐Centred Care for Older People in Long‐Term Care Facilities: A Mixed Methods Systematic Review

**DOI:** 10.1111/jan.70527

**Published:** 2026-02-15

**Authors:** Kedsaraporn Udkunta, Nikolaos Efstathiou, Ping Guo

**Affiliations:** ^1^ Department of Nursing and Midwifery, School of Health Sciences, College of Medicine and Health University of Birmingham Birmingham UK; ^2^ Department of Nursing Administration, Faculty of Nursing Chiang Mai University Chiang Mai Thailand

**Keywords:** long‐term care, long‐term care facilities, model of care, nurse, nursing, older people, person‐centred care, systematic review

## Abstract

**Aims:**

To critically appraise and synthesise the evidence about the effects and experiences of care models and interventions to improve person‐centred care for older people in long‐term care facilities.

**Design:**

A mixed methods systematic review, following the Joanna Briggs Institute guidance and the Preferred Reporting Items for Systematic Reviews and Meta‐Analyses.

**Data Sources:**

MEDLINE, PubMed, CINAHL, PsycINFO, Embase, Web of Science, Cochrane Library, and Thaijo were searched to identify relevant primary research published in English and Thai from January 2000 to February 2024.

**Review Methods:**

All relevant primary research with quantitative, qualitative, and mixed methods design was included. A convergent synthesis approach was used to synthesise and integrate findings.

**Results:**

4070 records were identified, of which 30 studies were retained: 12 quantitative, nine qualitative, and nine mixed methods studies. The evidence revealed five themes: (1) personalised preference, social engagement, and well‐being; (2) autonomy and dignity; (3) a home‐like environment; (4) family involvement and satisfaction; and (5) organisational and staff support.

**Conclusion:**

This review indicated that person‐centred care models and interventions could improve residents' quality of life, autonomy, and promote individual care provision, create an environment based on individual needs, and involve families in care, although challenges such as staff shortages and lack of managerial support may hinder successful implementation. Future work is required to evaluate and identify effective strategies to strengthen organisational support, including leadership development, staff retention, and resource allocation, and evaluate how organisational culture influences the adoption and success of person‐centred care practices.

**Impact:**

The review provides valuable insights and a comprehensive understanding of the care models and interventions specifically designed to improve person‐centred care and enhance the quality of life for older people in long‐term care facilities.

**Patient or Public Contribution:**

Not applicable.

**Trial Registration:**

The protocol was registered with the PROSPERO (CRD42024509504)

## 1 Introduction

The world's population of older people aged 65 years and older is projected to double in the next 30 years (from one billion in 2020 to 2.1 billion in 2050), and most of them will experience complex health problems (World Health Organization 2022). The prevalence of neurocognitive disorders such as dementia and Alzheimer's disease continues to increase (United Nations General Assembly 2013), and the common comorbid conditions among older people (e.g., hypertension, cardiovascular disease, stroke, arthritis, depression and functional disability) highlight the complexity of health challenges faced by this demographic (Moore et al. 2014). This trend contributes to a growing demand for long‐term care services, placing additional pressure on healthcare systems and caregiving resources (United Nations General Assembly 2013). The demand for long‐term care continues to rise yearly. The number of older people requiring long‐term care is projected to increase from 6.3 million in 2015 to approximately 15 million by 2050, which is driven by the desire of older people to reduce the burden on their families (Wu and Rong 2020; National Academy of Social Insurance 2025). Consequently, it is anticipated that there will be an increasing reliance on long‐term care to meet the needs of aging populations (National Academy of Social Insurance 2025).

However, the growing demand for long‐term care is straining existing systems, revealing critical gaps in service availability, workforce and quality of care (Spasova et al. 2018; Feng et al. 2020). Without targeted improvements in access, staffing, and regulatory oversight, the care provided to older people risks falling short of their complex needs (Spasova et al. 2018; Feng et al. 2020). These challenges, including limited infrastructure, staff shortages, and weak quality controls, not only hinder service delivery but also lead to substandard care, thereby adversely affecting quality of life and contributing to dissatisfaction with the overall standard of care (Spasova et al. 2018; Feng et al. 2020).

Evidence suggests that older people are not always satisfied with the quality of care provided in long‐term care due to unmet care needs and a lack of person‐centred care (Duan et al. 2020). They often experience loneliness, anxiety, psychological distress, strained relationships with healthcare providers, and inadequate communication (Tobis et al. 2021). Moreover, they face difficulties adjusting to their environment such as living in shared rooms, which often disrupts their sleep (Wu and Rong 2020). Their quality of life tends to be poor when they live in long‐term care facilities. Therefore, implementing person‐centred care that addresses the needs of older people in long‐term care is crucial for enhancing individuals' quality of life and improving healthcare services (American Geriatrics Society 2016).

Person‐centred care is defined as a method of providing care that considers the needs of patients, families, and communities and encourages patients and their families to participate in their health and work with healthcare providers to develop solutions that respond to their needs and preferences holistically (World Health Organization 2015; Health Innovation Network 2016). By fostering this inclusive and responsive model, person‐centred care plays a vital role in improving healthcare quality, raising service delivery standards, ensuring equitable access to care, promoting self‐care, and releasing the strain on healthcare and social service systems (Health Innovation Network 2016). It could also potentially improve job satisfaction and reduce emotional exhaustion in healthcare providers (Grevelink et al. 2012). Implementing a person‐centred care model for older people with dementia in residential aged care has shown clear benefits such as reducing reliance on antipsychotic and sedative medications, enhancing staff competence in dementia care, and fostering a supportive organisational culture (Roberts et al. 2015). Evidence also highlights broader impacts, greater family satisfaction, more responsive and flexible care, increased resident involvement, and improved overall well‐being (Chenoweth et al. 2015). These outcomes underscore the model's potential to transform aged care environments into more compassionate, effective, and empowering spaces for both residents and caregivers.

However, the majority of person‐centred care approaches have been developed and evaluated in high‐income countries, and they do not comprehensively represent all countries globally (Guist et al. 2020). This systematic review aims to evaluate the effects and experiences of care models and interventions to improve person‐centred care for older people in long‐term care facilities. Specifically, the review will identify and synthesise global evidence on the key components and effects of care models and interventions designed to improve person‐centred care for older people in long‐term care, and on the experiences and perceptions of older people, family caregivers, and healthcare providers regarding these care models and interventions.

## 2 The Review

### 2.1 The Review Questions

1. What are the key components and effects of care models and interventions to improve person‐centred care for older people in long‐term care facilities?
2. What are the perceptions and experiences of older people, family caregivers, or healthcare providers about care models and interventions to improve person‐centred care for older people in long‐term care facilities?

### 2.2 Methods

This systematic review followed the Joanna Briggs Institute (JBI) guidance and the Preferred Reporting Items for Systematic Reviews and Meta‐Analyses (PRISMA) to report a systematic review (Page et al. 2021). The review protocol was registered with PROSPERO. JBI guidance was applied to inform the design of the review method, while PRISMA was used to present the study selection process and report the findings (Page et al. 2021).

#### 2.2.1 Search Strategy

The search was performed using the following databases: MEDLINE, PubMed, CINAHL, PsycINFO, Embase, Web of Science, Cochrane Library and Thaijo. Each database is a well‐established multidisciplinary platform that contains indexed citations and abstracts on nursing, healthcare, medicine, and psychology (Bettany‐Saltikov 2016). The search was conducted using keywords and synonyms for all the component parts of the review question using the Population, Concept, and Context framework (PCC) and then combined them using Boolean operators (Bettany‐Saltikov 2016). The search terms are shown in Table 1.

**TABLE 1:** Search terms used.

Key concepts	Keywords
Older people (Population)	“older people” OR “elderly people” OR “older person*” OR “elderly person*” OR “aged” OR “aging population*” OR “elderly population*” OR “senior citizens”
Person‐centred care (Concept)	“person‐centred care” OR “person‐centered care” OR “individualised care” OR “individualised care” OR “personalise care” OR “personalised care” OR “integrated care”
Long‐term care facilities (Context)	“long‐term care” OR “long‐term care facilities” OR “long‐term care settings” OR “long‐term care institutions” OR “nursing homes” OR “residential care” OR “residential aged care” OR “care homes” OR “aged care homes” OR “home care services” OR “retirement homes”

#### 2.2.2 Eligibility Criteria

##### 2.2.2.1 Inclusion Criteria

Studies were included in the review if they met the following inclusion criteria:

1. Studies evaluating the effects or/and experiences of care models and interventions to improve person‐centred care for older people residing in long‐term care facilities (e.g., nursing homes, residential care homes, retirement homes).
2. Studies published in English or Thai from January 2000 to February 2024.
3. Studies with quantitative, qualitative or mixed methods designs.

##### 2.2.2.2 Exclusion Criteria

Studies were excluded from the review if they met the following exclusion criteria:

1. Studies that did not involve care models and interventions to improve person‐centred care for older people in long‐term care facilities
2. Secondary research (e.g., secondary analysis and reviews)
3. Editorials, expert opinions, conference proceedings and grey literature
4. Studies published in languages other than English or Thai and those published before 2000.

#### 2.2.3 Study Selection Process

Following the database searches, all identified citations were collated and imported into Covidence (Covidence is a systematic reviews production tool for title/abstract screening, full‐text screening, data abstraction, and quality assessment) to remove duplicates. Titles and abstracts were independently screened by two reviewers against predefined inclusion and exclusion criteria to identify and exclude irrelevant articles. The full texts of the remaining articles were retrieved, read, and assessed in detail against the inclusion criteria by two reviewers independently. Reasons for exclusion of full‐text studies were recorded and reported. Any differences that arose between the reviewers during the study selection process were resolved by discussion or through consultation with a third reviewer.

#### 2.2.4 Data Extraction

The first reviewer designed the data extraction table and performed the data extraction based on an existing template. Two additional reviewers from the research team subsequently reviewed the table to ensure accuracy and completeness. The data extracted included specific details about each paper: the author, year, country, aims, the participants and settings (including demographics, sample size, and geographical location), study designs, data collection and analysis methods (including outcomes and outcome measures), care models/interventions and comparisons if any, and key findings.

#### 2.2.5 Quality Assessment

All studies were critically evaluated by using the Mixed Method Appraisal Tool (MMAT) (Hong et al. 2018). Each study, whether qualitative, quantitative, or mixed methods, was evaluated according to specific criteria—five items for qualitative, five items for quantitative studies and 15 items for mixed methods studies. Each included study was appraised by the reviewers. Using MMAT involves three key steps. First, reviewers answered two initial screening questions to determine whether a study is empirical and therefore suitable for MMAT appraisal. Second, reviewers selected the appropriate study design category from five options (qualitative, randomised controlled, nonrandomised, quantitative descriptive, or mixed methods); mixed methods studies require appraisal across qualitative, quantitative, and mixed methods criteria. Third, the selected criteria were rated as Yes, No, or Can't tell, depending on whether the criterion is met or if insufficient information is provided. Quality scores were not calculated in accordance with the approach recommended by the developers of MMAT, which advises against presenting an overall score because it is not sufficiently informative when compared with a detailed descriptive summary based on the MMAT criteria (Hong et al. 2018). No studies were removed based on quality assessment due to their potential contributions to new knowledge and insights.

#### 2.2.6 Data Synthesis

A convergent synthesis approach was employed in this review (Hong et al. 2017). This includes quantitative and qualitative synthesis separately, followed by the integration of the resultant quantitative and qualitative evidence. A narrative synthesis was conducted to describe and compare the results of the quantitative studies. Extracted data were presented by textual description, tabulation, grouping, and clustering to identify the key components and effects of person‐centred care models and interventions for older people in long‐term care facilities.

A meta‐aggregation approach was used to analyse and aggregate qualitative findings from the perceptions and experiences of older people, family caregivers, or healthcare providers on care models and interventions to improve person‐centred care for older people in long‐term care facilities (Lockwood et al. 2015). Standard steps recommended by the Joanna Briggs Institute for meta‐aggregation were followed. Firstly, individual findings were evaluated, and one of three outcomes was recorded: unequivocal, credible, and unsupported. Then, unequivocal findings (defined as findings supported by incontrovertible evidence, where the findings could not be reasonably disputed) and credible findings (defined as findings based on plausible and coherent evidence, but where some degree of interpretation or challenge remained) were categorised according to meaningful similarity. For findings that captured several ideas, they were assigned to several matched categories according to the statements and illustrations. All the findings involved in categorising were original themes produced by the authors, with no changes. Finally, similar categories were synthesised to produce a single comprehensive synthesised finding. Then, findings from quantitative and qualitative evidence were integrated and presented in a thematic way.

## 3 Results

**FIGURE 1:**
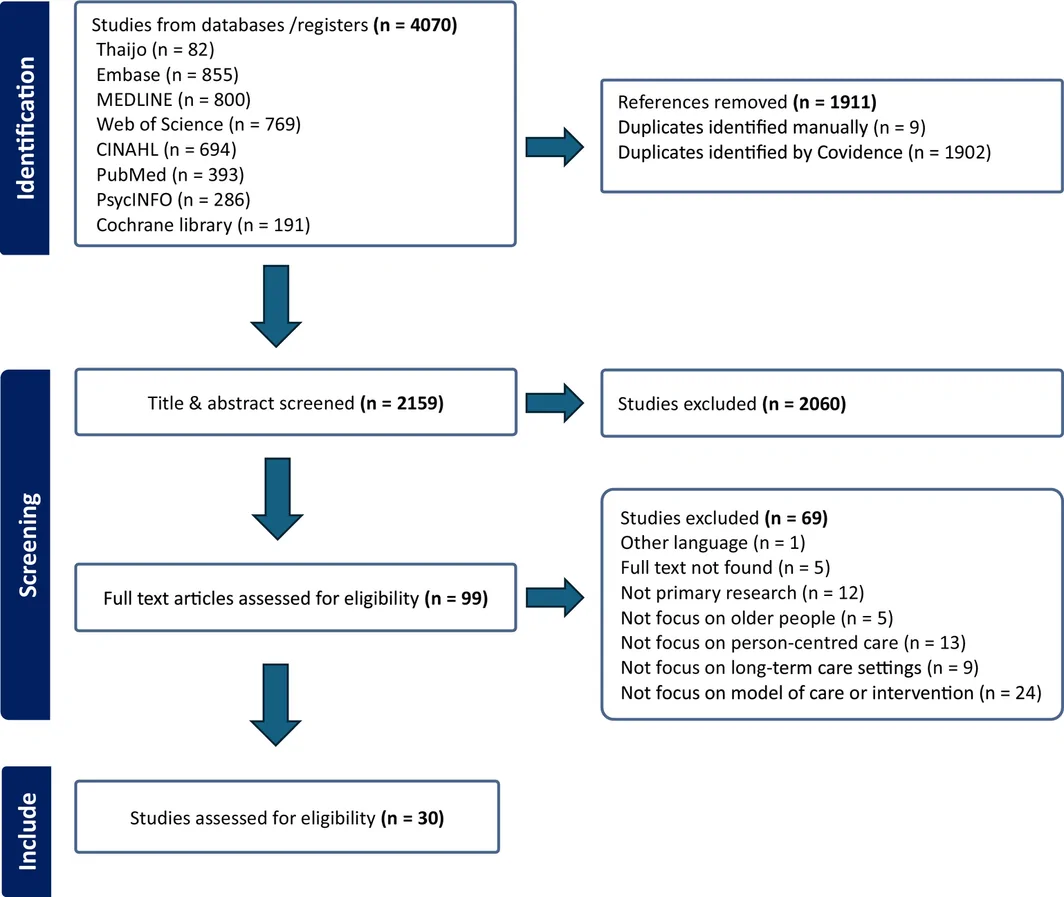
PRISMA flow diagram.

Following the database searches, 4070 potentially relevant articles were collated and imported into Covidence. 1911 duplicates were removed, and 2060 articles were excluded after the screening of titles and abstracts. Ninety‐nine full‐text articles were obtained through the search strategy and reviewed in further detail. After full‐text articles screening, 69 articles were excluded because they did not meet inclusion criteria. Finally, 30 studies were included in the review (Roberts et al. 2015; Chenoweth et al. 2015; Kim and You 2022; Kusmaul and Tucker 2020; Parkinson and Thompson 2022; Vassbø et al. 2019; Lynch et al. 2018; Donnelly and MacEntee 2016; Røsvik et al. 2011; Edvardsson et al. 2010, 2014; Pakkonen et al. 2023; Sjögren et al. 2022; Alexiou et al. 2021; Lood et al. 2020; Bökberg et al. 2019, 2020; Ballard et al. 2018; Barbosa et al. 2017, 2015; Hunter et al. 2016; Caspar et al. 2013; Coleman and Medvene 2013; Passalacqua and Harwood 2012; Choi et al. 2021; Jacobsen et al. 2017; Quasdorf et al. 2016; Taylor et al. 2015; Toit et al. 2020; Williams et al. 2015) (Figure 1).

### 3.1 Study Characteristics

This review retained twelve quantitative studies (Pakkonen et al. 2023; Sjögren et al. 2022; Alexiou et al. 2021; Lood et al. 2020; Bökberg et al. 2019; Ballard et al. 2018; Barbosa et al. 2017; Hunter et al. 2016; Edvardsson et al. 2014; Caspar et al. 2013; Coleman and Medvene 2013; Passalacqua and Harwood 2012), nine qualitative studies (Chenoweth et al. 2015; Kim and You 2022; Kusmaul and Tucker 2020; Parkinson and Thompson 2022; Vassbø et al. 2019; Lynch et al. 2018; Donnelly and MacEntee 2016; Røsvik et al. 2011; Edvardsson et al. 2010) and nine mixed method studies (Roberts et al. 2015; Barbosa et al. 2015; Bökberg et al. 2020; Choi et al. 2021; Jacobsen et al. 2017; Quasdorf et al. 2016; Taylor et al. 2015; Toit et al. 2020; Williams et al. 2015). The study characteristics are shown in Tables 2 and 3.

**TABLE 2:** Characteristics of included studies (quantitative and mixed‐method studies).

Author/Year/Country	Design	Aims/objectives	Participants/settings	Model or intervention/control	Data collection	Data analysis	Outcomes/results	Conclusion
Pakkonen et al. (2023), Finland	Quasi‐experimental cluster design with intervention and control groups.	To evaluate the effectiveness of a continuing education (CE) intervention named ‘Person First—Please’ (PFP) for improving nurses' PCC competence and its connection to PCC climate.	The professional nurses (*n* = 200) IG (*n* = 77): RNs or elderly care professionals (*n* = 13), LPNs (*n* = 64), average age 47.06 years, average working experience 8.45 years. CG (*n* = 123): RNs or elderly care professionals (*n* = 18), LPNs (*n* = 105). Average age 44.76 years, average working experience 5.38 years. Residents (*n* = 39) IG (*n* = 18): average age 86.89 years, average living time 1.79 years. CG (*n* = 21) participants. The average age was 86.43 years, average living time 2.62 years. 6 LTC settings for older people in two middle‐sized cities in western Finland.	IG: CE intervention (Continuing Education intervention, PFP), (10 weeks). CG: usual care.	Paper‐pencil questionnaires using PCC‐S and PCQ‐S pre‐intervention, after intervention immediately, and after the 6‐week intervention with nurses. Structured interviews with residents, supported by their next of kin at the same time points as the nurses' data collection, using the PCQ‐P to assess the person‐centred care climate.	Descriptive and inferential statistics	Primary: Professional nurses' PCC competence (PCC‐S) was changed for M0‐M2 when REML estimates of the change were from −0.29 (SE 0.05, Cl −0.42, −0.17) to −0.36 (SE 0.07, Cl −0.52, −0.20). Secondary: The PCC climate (PCQ‐S) was changed for M0‐M2 when REML estimates of the change were from −0.48 (SE 0.08, Cl −0.36, −0.01) to 0.48 (SE 0.09, Cl −0.67, −0.29). Resident: The PCC climate (PCQ‐P) was changed for M0‐M2 when REML estimates of the change were −0.35 (SE 0.13, Cl −0.66, −0.03)	The intervention enables increased PCC competence of nurses and increases the PCC climate for both nurses and residents with their next of kin.
Sjögren et al. (2022), Australia, Norway and Sweden.	A multi‐centre, non‐equivalent controlled group before‐after design.	To evaluate the effects of a person‐centred and thriving‐promoting intervention on nursing home residents´ experiences of thriving and person‐centredness of the environment, and to evaluate if the effects varied between female and male residents.	The residents in IG: T0 (*n* = 139), T1 (*n* = 114), T2 (*n* = 114) CG: T0 (*n* = 153), T1 (*n* = 138), T2 (*n* = 91). Mean ages 85.9, 85.7, and 86.7 years, respectively, female (74%, 71%, and 72%, respectively) 6 Nursing homes in Australia, Norway, and Sweden.	IG: education programme on the theoretical understanding of person‐centredness, person‐centred care, thriving and caring environment over a 14‐month period. CG: a two‐hour introduction lecture.	The questionnaires used included demographic variables and instruments for measuring the residents’ independence in ADL, cognitive capacity, the prevalence of neuropsychiatric symptoms, and PCQ‐P. Proxy‐assessments by staff were conducted due to the expectedly large group of residents with cognitive impairment in NHs before the intervention started, immediately after the intervention, and 6 months after the intervention was completed.	Descriptive statistics and linear regression model.	After intervention: The thriving score increased between T0‐T1 (mean difference 4.2) and between T1‐T2 (mean difference 0.5). Statistically significant effect on TOPAS for male residents between T0 and T2 (mean change 24.6, *p* = 0.001, Partial Eta Squared 0.06). The person‐centredness climate (PCQ‐P) score increased between T0‐T1 (mean difference 4.3) and between T1‐T2 (mean difference 0.3).	The intervention affects residents’ thriving and person‐centredness of the environment, particularly with significant effects on the male residents’ perceptions.
Alexiou et al. (2021), Sweden	A controlled prospective cohort study.	To focus on the impact of the implementation of a person‐centred approach on staff perception of the likelihood of being able to provide person‐centred care and strain in the workplace.	Nursing staff members (*n* = 92): IG (*n* = 54), CG (*n* = 38): men (7.2%), aged 28‐57 years, mean age 45.29 years. 3 RCFs in western Sweden (two intervention sites and one control site).	IG: the person‐centred approach: staff‐resident encounters, integrating the essence of the resident's narrative into the care plan and evaluating the outcome. CG: usual care.	The questionnaires used included the PCQ‐S, P‐CAT, and SNCS before implementing a person‐centred approach (T0), during implementation (T1), immediately after the end of the implementation programme (T2), and 6 months later (T3) prospectively from 2013 to 2015.	Descriptive statistics, the analysis of variance tool (ANOVA), fisher's test, and t‐test	After an intervention, the PCQ‐S score showed no significant changes (mean score of T0 = 4.78, T1 = 4.82, T2 = 4.88, T3 = 4.81). The P‐CAT score showed no significant changes (mean score of T0 = 3.72, T1 = 3.83, T2 = 3.83, T3 = 3.86). SNCS score showed no significant changes (mean score of T0 = 1.90, T1 = 1.93, T2 = 1.81, T3 = 1.91). A weak negative correlation was found between P‐CAT‐ scores and nursing staff age (*r* = −0.17, *p* ≤ 0.05). The weak positive correlation between SNCS scores and nursing staff age (*r* = 0.17, *p* ≤ 0.05).	The intervention had no effect on person‐centred care, whereas older ages and long durations of work experience significantly negatively affected the staff's assessment of their ability to create an atmosphere of everydayness and to adopt a person‐centred approach in care.
Lood et al. (2020), Australia, Norway and Sweden.	A multi‐centre, non‐equivalent controlled group before‐after design.	(1) to evaluate the effects of a staff education programme about person‐centred care and promotion of thriving on relatives’ satisfaction with the quality of care and their perceptions of the person‐centredness of the environment. (2) to outline factors of importance to explain the variance in relatives’ satisfaction with quality of care.	The relatives of older people (*n* = 459): IG: T0 (*n* = 91), T1 (*n* = 80), T2 (*n* = 79) CG: T0 (*n* = 87), T1 (*n* = 69), T2 (*n* = 53) mean age 64.7 years, women (64%), children to the person living in the nursing home (68%), and visited the nursing home every week (68%). 6 Nursing homes in Australia, Norway and Sweden.	IG: education programme on the theoretical understanding of person‐centredness, person‐centred care, thriving and caring environment over a 14‐month period. CG: one‐hour lecture before the intervention period.	The questionnaires used included questions on the relatives’ background characteristics, questions on their satisfaction with the quality of care, and PCQ‐F at baseline, after the intervention, and 6 months after the end of the intervention.	Descriptive statistics, t‐test, Mann‐Whitney *U*‐test, or Chi‐square, a generalised linear model and hierarchical multiple regression.	The relatives in the CG at T0 were more satisfied with the quality of caring processes than IG (*p* = 0.01), and T2 were more satisfied with the quality of information (*p* = 0.02) and nursing staff (*p* = 0.03) than IG. The relatives in the IG were satisfied with the quality of the work environment (*p* = 0.03). Safety had a statistically significant association with the relatives’ satisfaction with the quality of care (*β* = 0.67, *p* = 0.01). Hospitality had a strong and statistically significant association with the relatives’ satisfaction with the quality of care (β = 0.32, *p* = 0.01).	The relatives from both the intervention and control nursing homes were satisfied with the quality of care, particularly in terms of safety and hospitality, which were identified as factors of prominent importance for the relatives’ satisfaction with the quality of care.
Bökberg et al. (2019), Sweden	Pre‐post testing experimental design	To evaluate whether an educational intervention had any effect on the staff's perception of providing person‐centred palliative care for older persons in nursing homes.	Staff members (*n* = 365): IG (*n* = 167), CG (*n* = 198) AN (*n* = 330), RN (*n* = 16), occupational therapist (*n* = 4), physiotherapist (*n* = 4), frontline manager (*n* = 11) median range ages 21–66 (IG) and 21–65 (CG), women (*n* = 157 in IG and *n* = 189 in CG). 20 nursing homes in Sweden.	IG: the knowledge‐based palliative care intervention (9‐month period). CG: usual care.	The questionnaires used included the PCQ‐S and P‐CAT at baseline (before intervention) and 3 months after (follow‐up) the educational intervention end.	Descriptive statistics, Wilcoxon signed rank test, Pearson chi‐square test or Fisher's exact test, Mann‐Whitney *U*‐test and the Kruskal‐Wallis test, and A univariate logistic regression analysis.	The median P‐CAT scores in IG and CG at baseline were 45 and 44, respectively (*p* = 0.601). The median P‐CAT scores at follow‐up were 44 in both the IG and CG (*p* = 0.601). The median PCQ‐S scores at baseline were 77 in both the IG and CG (*p* = 0.451). The median PCQ‐S scores in IG and CG at follow‐up were 71 and 74, respectively (*p* = 0.451). The median score of organisational and environmental support in IG and CG both at baseline and follow‐up was 11 (*p* = 0.237).	Both the intervention group and the control group revealed high median scores in all subscales at baseline, except for the subscale amount of organisational and environmental support in the P‐CAT.
Ballard et al. (2018), UK	A randomised controlled cluster trial.	To evaluate the efficacy of a person‐centred care and psychosocial intervention incorporating an antipsychotic review, WHELD, on QoL, agitation, and antipsychotic use in people with dementia living in nursing homes, and to determine its cost.	The residents with moderately severe or severe dementia (*n* = 847) IG (baseline cohort = 404, completer = 257) CG (baseline cohort = 443, completer = 296) 69 UK nursing homes.	IG: the WHELD intervention in a 9‐month clinical trial. CG: treatment as usual (TAU).	The questionnaires used included the DEMQOL‐Proxy to assess quality of life (QoL), the CMAI to assess agitation, and the NPI‐NH to assess neuropsychiatric symptoms, mood, antipsychotic use, unmet needs, quality of interactions, pain, mortality, and cost, all of which were assessed by a trained research assistant prior to randomisation and again after 9 months of the intervention.	A multilevel analysis of covariance (ANCOVA).	The WHELD intervention had statistically significant benefits in: The quality improvement (DEMQOL‐Proxy Z score 2.82, *p* = 0.0042). Agitation (CMAI Z score 2.68, *p* = 0.0076) Neuropsychiatric symptoms (NPI‐NH Z score 3.52, *p* < 0.001). Positive care interactions (19.7% increase).	The WHELD intervention provides benefits in terms of quality of life, reduced agitation, and improvements in neuropsychiatric symptoms, though the effect sizes are relatively modest. Additionally, it offers cost savings and can be easily implemented in nursing homes.
Barbosa et al. (2017), Portugal	An experimental study used a controlled pre‐post testing design.	To assess the effects of a psycho‐educational (PE) programme on the quality of DCWs’ interactions with residents with dementia.	DCWs (*n* = 58): EG (*n* = 27), CG (*n* = 31): female (*n* = 58), mean age of 44.72+‐ 9.02 years, married (67.2%), primary and middle school education (46.4%), and high school (41.4%), average length of service 9.61+‐3.72 years. 4 aged‐care facilities.	EG: PCC‐based psycho‐educational intervention. CG: PCC‐based education‐only intervention.	DCWs’ socio‐demographic data were collected through a structured questionnaire. DCWs’ person‐centredness, and morning care interactions data were video recorded at baseline and 2 weeks after the intervention and using the GBS to code. Recordings started at the moment the DCWs entered the room and stopped when they left.	Descriptive statistics, *t*‐test, and ANOVA.	Both groups reported significantly higher scores on eight of 11 items of the GBS and on the GBS total score at post‐test (*F* = 10.59; *p* = 0.02). GBS total score improvements were higher for the EG, with values close to significance (*F* = 3.90; *p* = 0.054).	A psycho‐educational intervention may enhance care workers' person‐centredness.
Hunter et al. (2016) Canada	Correlational study	To investigate the association of personal and organisational environmental characteristics with self‐reported person‐centred behaviours in long‐term residential care settings.	Staff members (*n* = 108): Nurses (25%), NAs (40.7%), worked in areas (34.3%) (administration, recreation, housekeeping, and dietary), males (*n* = 11), females (*n* = 97), mean age 43.5 years. 2 large rural long‐term residential care facilities in Western Canada.	None	The questionnaires used included the Person‐Directed Care scale, Self‐reported person‐centred care, organisational‐environmental supports for person‐centred care, Beliefs about personhood in dementia, and burnout.	Multiple linear regression analysis and changes in R2 to test variable associations.	Person‐directed environment has a statistically significant positive association with autonomy (*p* < 0.05). Support for work with residents has a statistically significant positive association with attention to personhood (*p* < 0.05), knowledge of residents (*p* < 0.05), and comfort care (*p* < 0.05). Both beliefs about personhood in dementia and personal accomplishment, an aspect of burnout are statistically significant positive associations with attention to personhood (*p* < 0.05).	Organisational characteristics influence various aspects of person‐centred dementia care. However, individual characteristics such as gender, beliefs about personhood, and burnout seem to play a more significant role in certain elements of PCC.
Edvardsson et al. (2014), Sweden	A quasi‐experimental, one group pre‐post testing design.	To evaluate the effects of implementing national guidelines for person‐centred care of people with dementia on self‐reported person‐centredness, strain, and stress of conscience as perceived by care staff.	Staff members (at baseline = 171, at follow‐up = 143) At baseline: female (84%), assistant nurse (42%), mean work experience 21 years in healthcare, 19 years in aged care, and 9 years in the current unit. At follow‐up: female (86%), assistant nurse (55%), mean work experience of 19 years in healthcare, 19 years in aged care and 9 years in the current unit. Swedish residential aged care facility located in the metropolitan Stockholm area.	Interactive stepwise program of knowledge translation, knowledge generation, and knowledge dissemination based on and the Swedish national guidelines for care of people with dementia (12‐month period).	The questionnaires used included PCAT, PCQ‐S, and staff job strain and stress of conscience at baseline and follow‐up by the staff self‐reported.	Descriptive statistics and the paired samples t‐test.	After the intervention: The staff scores on the person‐centredness of care were significantly increased (*p* < 0.01, Cohen's *d* = 0.34). The staff scores on stress of conscience were significantly reduced (*p* < 0.01, Cohen's *d* = 0.38).	The intervention increased the staff self‐reported person‐centeredness of care practice, perceived hospitality of the setting, and reduced staff stress of conscience.
Caspar et al. (2013), Canada	Correlational study	To explore the relationship between contextual‐level characteristics, individual‐level characteristics, and access to empowerment structures on care staffs’ perceived ability to provide I‐Care.	RNs (*n* = 176), LPNs (*n* = 65), care aides (*n* = 326): median range ages of RNs/LPNs and care aides 19‐65 and 22‐64, women (95% of RNs/LPNs and 91.7% of care aides). 41 LTC facilities within three of five regional health authorities in British Columbia, Canada.	None	All participants received a survey package that contained the study information document and the study questionnaires consisting of a demographic questionnaire, CWEQ, JAS, ORS, and the I‐Care instrument. Then, the participants answered the questions and returned them to the primary investigator.	Multilevel model.	91.7% of the total variance in perceived ability to provide I‐Care reflected within‐ versus between‐person differences, with the 6 empowerment variables accounting for 31% of this within‐person variance independent of the other context‐ and person‐level covariates.	Contextual‐ and individual‐level factors exert considerably less influence on I‐Care than factors associated with staffs’ perceptions of empowerment.
Coleman and Medvene (2013), USA	A quasi‐experimental, wait‐list control design.	To further define the interpersonal aspects of person‐centred care by developing and testing an intervention to promote the interpersonal aspects of person‐centred care.	Residents (*n* = 12): mean ages in NH1 and NH2 82.6 and 86.3 years, respectively, female (NH1 = 50%, NH2 = 83.3%), white (NH1 = 91.7%, NH2 = 100%). CNAs (*n* = 12): mean ages of residents in NH1 and NH2 32.1 and 30.8 years, respectively, female (NH1 = 66.7%, NH2 = 83.3%), black (68.7% in NH1), white (91.7% in NH2) Nursing home from a private Midwestern healthcare system.	The treatment group in NH1 (*n* = 12 dyads): the Person‐Centred Care Intervention (3‐month period). Control group in NH2 (*n* = 12 dyads): routine care. The intervention was conducted with the control group after the treatment group for 6 weeks.	After the training sessions, the trainers at each of the sites observed and documented the CNAs’ behaviour on the job using video recordings of the morning care, afternoon check‐ins, and physical rehabilitation. The videos at the NH1 site ranged from 30 s to 14.5min and at the NH2 site videos ranged from 1 to 13.5 min. Then, two observational instruments (GBS and PCBI) were used to code the extent to which the CNA provided care in a person‐centred way.	Wilcoxon signed ranks test.	After the intervention, the resident's perceptions of relationship closeness increased significantly at both NHs, NH1, *z* = −1.89, *p* < 0.05, and NH2, *z* = −1.95, *p* < 0.05.	The intervention affects the CNA's perceptions of satisfaction and closeness and resident satisfaction.
Passalacqua and Harwood (2012), USA	An experimental with pre‐post testing design.	To test the feasibility of a series of workshops built around VIPS, intended to increase the person‐centred communication, beliefs, and attitudes among paraprofessional dementia caregivers in a long‐term care facility.	Caregivers (*n* = 26): female (89%); 18–30 years (46%), 31–49 years (27%), 50 years (27%), South or East Asian (35%), Hispanic (35%), White (15%), and Black (15%). A for‐profit long‐term care facility in the US Southwest that specialises in memory issues.	VIPS Communication Skills Intervention. (over a period of 4 weeks). Pre‐ and post‐test in the same group.	Questionnaires were completed at monthly staff meetings, one of which occurred 4 weeks before the beginning of the intervention, and the second of which occurred about 6 weeks after the end of the intervention. The questionnaires consisted of empathy, happiness, burnout, attitude about aging, and quality communication.	ANCOVA test.	After the intervention: Less depersonalisation of resident from 1.71 to 1.16 (*p* < 0.05). More hope for Alzheimer's patients from 2.79 to 4.48 (*p* < 0.01). More empathy from 4.18 to 4.50 (*p* < 0.10). Using more gestures from 3.52 to 4.16 (*p* < 005). Using more humour from 3.58 to 4.04 (*p* < 0.10). Asking more yes/no questions from 3.92 to 4.36 (*p* < 0.05). Giving a choice between two options more from 4.12 to 4.48 (*p* < 0.05).	The intervention is a useful tool to improve the quality of dementia care provided by paraprofessional dementia caregivers in a long‐term care facility.
Choi et al. (2021), Korea.	A mixed‐methods design	To evaluate the implementation fidelity of the SPEC intervention and to identify moderating factors that influence the implementation fidelity.	Interdisciplinary team (*n* = 20) 10 NHs in Seoul, South Korea.	The System for Person‐Centred Elder Care (SPEC) intervention in a 6‐month period.	SPEC consultants' logbooks, the SPEC ICT system, and a standardised, self‐reported paper were collected from coordinators before, during, and after the 6‐month intervention phase. Qualitative data: SPEC consultants' logbooks, a semi‐structured questionnaire with free‐text responses, and focus group interviews with each nursing home's interdisciplinary team, lasted 1 h and after the 6‐month intervention period.	Quantitative: descriptive statistics Qualitative: content analysis	65% of on‐site coordinators reported that the SPEC model positively impacted the needs assessment and reporting system for resident care, and 75% of them would recommend the SPEC model to other NHs. 6 domains and 21 themes: Participant responsiveness: boosting CGA, facilitating communication, providing tailored care plan, duplication of using SPEC system, lack of staffing level and time. Intervention complexity: comprehensiveness of intervention description, complexity of interventions, difficulty of using ICT system. Facilitating strategies: provision of immediate feedback, tailored consulting and extra education. Quality of delivery: well‐prepared training and manuals, provision of individual/institutional report, reflection of preferences of participants, needs for practical system for Korean NHs. Recruitment: recruiting a proactive NH head, presentation for key care team for participation, difficulty of maintaining participation due to workload. Context: supportive or individualistic organisational culture, supportive leadership, current events.	The SPEC program was good implementation fidelity, and strong in all aspects The important facilitating factors were tailored facilitating strategies, assurance of the quality of delivery, and recruitment strategies.
Bökberg et al. (2020), Sweden.	Convergent mixed method (a crossover design)	To evaluate person‐centredness in nursing homes from the perspective of frail older persons, before and after implementing an educational intervention about palliative care.	Older persons living in nursing homes (*n* = 44): IG (*n* = 24), CG (*n* = 20) 20 nursing homes in two different counties (rural and urban) in southern Sweden.	IG: the Knowledge‐based palliative care intervention a (6‐month period) (interviewed at both baseline and follow‐up 3 months after intervention)	The questionnaires used included PCAT and PCQ‐P, both quantitative and qualitative before the intervention started and followed up 3 months after the intervention was completed. In the structured interviews, the older persons expressed their experiences of the care related to person‐centeredness, which lasted an average of 45–60 min.	Quantitative: Wilcoxon signed‐rank test, Mann‐Whitney *U* test, Pearson Chi‐square test or Fisher's exact test. Qualitative: content analysis	The median score before and after intervention in the intervention group. Personalising care: 24.5 and 25.0 (*p* = 0.659). Organisational and environment support: 11.0 and 10.0 (*p* = 0.887). Safety: 55.0 and 51.5 (*p* = 0.018). Everydayness: 19.5 and 20.0 (*p* = 0.563). Hospitality: 15.0 and 16.0 (*p* = 0.368). The older persons’ experiences expressed in the category Routines first then the older person, putting demands on next of kin, not feeling safe related to shortcomings in the staff, being able to take part in meaningful activities and social interaction, and the importance of a familiar environment.	The older people expressed feelings of unsafety related to shortcomings in staff.
Toit et al. (2020), Australia.	An embedded mixed methods study design.	To identify what particular aspects of the PEP these facilitators valued for advancing PCC and care culture change.	Dementia care specialists (*n* = 9) Care managers and RNs (attendees) Residential aged care facilities belonging to a nationwide care organisation.	The Peer Enablement Program (PEP). Facilitators conducted 3 workshops in nine sites.	Workshop evaluation forms aimed to measure learning objectives achieved and explore the insight and experiences of attendees collected after each workshop. Documents generated by the nine facilitators to prepare and deliver PEP workshops (Meeting minutes, WhatsApp) and reflection forms. A group discussion using the NGT; the question asked of these facilitators was, “In your opinion, what value did the PEP add to the advancement of PCC of older persons with moderate to advanced dementia?”	Quantitative: descriptive statistics. Qualitative: thematic analysis.	91.4% appreciate that behaviour is an expression of a person's lived experience. 90.6% improve the ability to distinguish between delirium and dementia. 87.8% improve skills in observing and assessing pain in a person with dementia. 86.8% identify and develop transformational leadership skills. Personal insights gained: 14.7% facilitating group person‐centred problem‐solving and new knowledge about delirium and the use of antipsychotic drugs (13.1% and 12.5%, respectively). The program facilitates collaboration among attendees, fosters the development of their professional identities as transformational leaders, and establishes communities of practice with the potential to sustain progress in person‐centred care.	Peer support is the most valued aspect of PCC.
Jacobsen et al. (2017), Norway	A mixed‐method design	To investigate which factors hindered or facilitated staff awareness related to confidence building initiatives based on PCC, as an alternative to restraint in residents with dementia in nursing homes.	Staff members (*n* = 452): 50% full‐time position, 39.6% RNs, 54.9%, auxiliary nurses, and 5.4% care assistants. 24 nursing homes in Western Norway.	IG (12 NHs): the MEDCED education intervention consists of a two‐day seminar and monthly coaching sessions for 6 months. CG (12 NHs).	Questionnaires used included PCAT and QPS‐Nordic forms, specifically focusing on the leadership dimensions. Focus group with facilitators took place before (FG 1–3) and during (FG‐4) the intervention. The ethnographic fieldwork was performed after the intervention, in six nursing homes in the intervention group. The fieldwork combined participant observation, and formal and informal interviews with staff, where observations contributed to formal and informal questions asked. 84 reflection notes from eight persons in the four teams who facilitated the intervention were analysed.	Quantitative: descriptive statistics. Qualitative: thematic analysis.	After intervention, the P‐CAT score slightly increased from 46.7 to 47.4 (*p* = 0.192). 13% of P‐CAT variation was explained by institutional belonging. The staff achieved an increased awareness with regard to person‐centred care, and more specifically, to a variety of confidence‐building measures as alternatives to use of restraints. The leaders closest to the decision‐making process played a pivotal role with regard to the potential success or failure of the intervention: increase the presence of the general staff at the supervision session, enhance the employment of agreed alternatives to restraint.	The interaction between leadership and staff culture emerged as the key factor influencing whether staff awareness of confidence‐building initiatives, rooted in person‐centred care, was hindered or promoted.
Quasdorf et al. (2016), Germany	A convergent parallel mixed‐methods design embedded in a quasi‐experimental trial	To evaluate Dementia Care Mapping implementation in nursing homes.	Head nurses (*n* = 9), nurses (*n* = 9), project coordinators (employees in NH) (*n* = 27), caregivers (*n* = 12). 9 nursing units in nine different nursing homes in north‐west Germany.	IG (units 1–3 = group A; units 4–6 = group B): Dementia Care Mapping CG (units 7–9 = group C): the regular and standardised quality assessment rating QUALIDEM.	Semi‐structured interviews with head nurses (T0, T2) and staff nurses (T1). Documents were collected throughout the study to reconstruct the implementation process. A random sample of three resident records was collected at each data collection time point and reviewed for PCC indicators. The Dementia Milieu Assessment (DMA) was used to assess the dementia friendliness of the milieu (T0, T2) (a 2‐h observation). Organisational characteristics using DIQ. A staff questionnaire assessing the fidelity dimensions of participant responsiveness and quality of programme delivery (T2).	Quantitative: descriptive statistics. Qualitative: Content analysis.	The essential factors influencing the implementation process include well‐functioning networks, a dementia‐friendly culture and Flexible organisational structures, individuals’ positive attitudes, planning the intervention, and recruitment (well‐qualified and experienced project coordinators). The challenges: networks and communication, staff turnover, changes in the head nurse or managers, and conflicts and misunderstandings within the teams.	For successful Dementia Care Mapping implementation, it must be embedded in a systematic implementation strategy considering the specific setting.
Barbosa et al. (2015), Portugal.	A mixed methods study	To analyse the effects of a PCC‐based psychoeducational (PE) intervention on DCWs’ work‐related stress, burnout, and job satisfaction.	DCW (*n* = 58): EG (*n* = 27), CG (*n* = 31): females (*n* = 58), mean age 44.72 ± 9.02 years, married (67.2%), primary and middle school (46.4%), high school (41.4%), the average length of service 9.61 ± 3.72 years. 4 aged‐care residential facilities in the central region of Portugal.	EG: a PCC‐based psychoeducational (PE) intervention (an intervention offering both educational and emotional support (2‐month period)). CG: education only.	Questionnaires used included a sociodemographic questionnaire, burnout, job satisfaction, and stress assessments, 2 weeks before and 2 weeks after the intervention. 8 focus groups (two per facility), each involving 5 to 12 participants, in a private and quiet room within the facility. Each session lasted around 45 minutes. The interview aimed to collect DCWs’ perceptions about the intervention and its impact on their working life.	Quantitative: descriptive statistics, *t*‐test, and chi‐square test. Qualitative: Thematic analysis.	Post‐test EG versus CG: Emotional exhaustion: 14.88 versus 15.42 (*p* = 0.029). Stress: positive but no significant effects (*p* = 0.826). Job satisfaction: positive change in EG whereas minimal change in CG (*p* = 0.618) Qualitative: the EG perceived enhanced group cohesion, better emotional management, and self‐care awareness Four themes were shared between the groups: self‐worth feelings, increased knowledge about dementia, increased knowledge about the person, and PCC awareness. The interventions improved DCWs’ competences on PCC, enhanced their attitude and commitment to work, and allowing DCW to better understand the residents. Hindering factors, workload, as a result of the lack of time and the shortage of staff and missed collaboration from their managers and reported feeling unappreciated.	Psychoeducational interventions may contribute to reduce direct care workers’ burnout.
Roberts et al. (2015), Australia	A mixed methods evaluation design	To describe the development of a composite model of care and its impact on people with dementia and report on the evaluation and results of a pilot project exploring the new model's feasibility.	Residents (*n* = 16): average age 85 years, females (*n* = 12), males (*n* = 4). Care staff (*n* = 18) Visiting families (*n* = 15) Residential age care in Australia.	The ABLE model features Montessori‐based activities during the 18‐month period, once at baseline or admission to the unit (pre‐test), and again at follow‐up (post‐test).	Data were collected at 2 time points during an 18‐month period, once at baseline or admission to the unit, and again at follow‐up. Residents’ use of antipsychotics or sedatives was collected through a retrospective audit of medication charts. Residents’ frequency of behavioural and psychological symptoms of dementia was measured by the 29 CMAI score. Qualitative interviews were conducted with family carer relatives to elicit their responses to the changes throughout the implementation period. Written qualitative comments from family members were collected on the RESI Resident/Relative Audit tools to capture their views about the Unit and the impact of the person‐centred intervention. TURNIP surveys were conducted with staff.	Quantitative: PASW 18.0. Qualitative: Thematic analysis.	After intervention: Anti‐psychotic and sedative medication used reduced from 75% to 16.66%. Physically non‐aggressive behaviour and verbally agitated behaviour there were significant reductions (physically non‐aggressive behaviour: *t* = 6.873, df =14, *p* < 0.001; verbally agitated behaviour, *t* = 6.632, df = 14, *p* < 0.001). Staffs’ knowledge about meeting the needs of people with dementia increased (*t* = 3.00, df = 26, *p* < 0.01). Organisational culture change that supported the ABLE model of care (*t* = 2.07, df = 26, *p* < 0.05). Families’ satisfied changes. The staff was overwhelmingly positive about the change: better‐supporting residents to express their identity, better supporting personal choices, and facilitated movement around the Unit, and they focused on quality interaction with residents. The organisation was aware of the importance of a person‐centred approach.	The intervention facilitates a reduction in the use of antipsychotic and sedative medications and physically non‐aggressive and verbally agitated behaviours and enhances both staff knowledge and family satisfaction.
Taylor et al. (2015), Australia	A convergent mixed methods study design	To evaluate the feasibility of an intervention to improve person‐centred mobility care during resident transfers.	Residents (*n* = 12) (unit A = 6, B = 2, C = 4): a mean age of 83 (SD 5), males (*n* = 5), females (*n* = 7) Direct care staff (*n* = 51): males (*n* = 9), females (*n* = 42), nurse supervisor (*n* = 9), carer (*n* = 25), MH trainer (*n* = 5), lifestyle staff (*n* = 5), nurse (*n* = 4), manager (*n* = 2), physiotherapist (*n* = 1) 3 resident aged care (2 high‐care units and one high‐care dementia‐specific) in Melbourne, Australia.	A collaborative training program was conducted over sixteen weeks.	Staff measurements included satisfaction with training and the program, knowledge tests, and mobility care during transfers (transfer observation tools). Resident measurements included the Physical Mobility Scale, degree of autonomy during transfers, and their experience of mobility care (transfer observation tools). Focus groups focused on deeper insights and audio recorded.	Quantitative: descriptive statistics, *t*‐test, and Manne Whitney. Qualitative: Thematic analysis.	After the training programme: 100% of staff satisfaction (40% rated excellent and 60% rated good). Staff's knowledge improved from 41% to 58% (*p* = 0.0005). Mobility care categorised as positive increased by 22% and negative instances of mobility care decreased by 26% (*p* = 0.0148). The residents’ autonomy and control during and experience of mobility care were improved. Two themes: the change context and mechanism of changes.	The program facilitates improving staff's knowledge of mobility care and residents’ autonomy and providing safe and quality care.
Williams et al. (2015), Canada.	A mixed‐methods research design	To examine a PCC programme implemented in three Canadian long‐term care facilities to determine its effect on resident outcomes, approach to care and maintenance of the programme 3 years after implementation.	Residents in the intervention Facilities (*n* = 206) Residents in the comparison Facilities (*n* = 182) RNs (*n* = 7), Registered Psychiatric Nurses (*n* = 3), LPNs (*n* = 6), RCC (*n* = 3), care aides (*n* = 22), inter‐facility managers (*n* = 2): women (*n* = 40), men (*n* = 3), mean age 45.19 years, experience in their current facility 9.72 years and LTC 14.35 years. 6 Canadian long‐term care facilities.	Intervention Facilities A, B and C: a person‐centred care programme training. Comparison Facilities: no training PCC programme.	Questionnaires used included the RAI‐MDS 2.0 which is a standardised measure designed for LTC which provides a comprehensive assessment of a resident's strengths, needs, concerns, and potential risks with residents before, after, and 6 months. 8 group interviews and 7 individual interviews: focus groups included 3–7 participants, with separate sessions held for nurses and care aides; the questions focused on general issues related to the culture of the care environment and philosophy of care and information about the functioning of the PCC programme.	Quantitative: Analyses of Covariance (ANCOVA). Qualitative: Thematic analysis with deductive framework.	After intervention EG vs. CG: The ISE scale: CG is higher than EG (*p* < 0.05), indicating more social engagement. The CHESS scale: EG is higher than CG (*p* < 0.05), indicating higher medical instability. The pain scale: EG is higher than CG (*p* < 0.05). There are no consistent differences in the care approaches of staff between the program and control facilities. Staff perceptions: their concerns related to problematic resident behaviours especially associated with dementia, and described the importance of assessment in determining causes and identifying potential solutions for these difficulties. All facilities supported elements of a person‐centred philosophy of care to reduce problematic behaviours and improve quality of life.	The intervention does not affect resident outcomes.

Abbreviations: CG, control group; CHESS, Changes in Health End‐stage Disease and Symptoms and Signs; CMAI, Cohen Mansfield Agitation Inventory; CNAs, certified nursing assistant; CWEQ, Conditions of Work Effectiveness Questionnaire; DCW, direct care worker; DIQ, the Dementia Institution Questionnaire; EG, Experiment group; FG, focus groups; GBS, Global Behaviour Scale; I‐care, Individual‐care; IG, intervention group; ISE scale, Index of Engagement Scale; JAS, the Job Activities Scale; LPNs, licensed practical nurses; LTC, long‐term care; NGT, nominal group technique; NH, nursing home; ORS, the Organisational Relationships Scale; P‐CAT, the Person‐centred Care Assessment Tool; PPC, person‐centred care; PCC‐S, person‐centred care tool‐staff version; PCQ, the Person‐Centred Climate Questionnaire; PCQ‐P, the Person‐centred Climate Questionnaire ‐ patient version; PCQ‐S, the Person‐centred Climate Questionnaire—Staff version; QoL, quality of life; RAI‐MDS, the Resident Assessment Instrument Minimum Data Set; RCC, Resident Care Coordinators who are also nurses; RCFs, residential care facilities; REML, estimates of the change; RNs, registered nurses; T0, at baseline; T1, first follow‐up; T2, second follow‐up; T3, third follow‐up; TAU, treatment as usual; TOPAS, Thriving of Older People Assessment Scale; TURNIP, Tool for Understanding Residents’ Needs as Individual Persons; VIPS, Valuing people, Individualised care, Personal perspectives, and Social environment.

**TABLE 3:** Characteristics of included studies (qualitative studies).

Author/Year/Country	Design	Aims/objectives	Participants/settings	Data collection	Data analysis	Findings
Kim and You (2022), Korea	Qualitative (Phenomenological method)	To explore the person‐centred care experienced by families of elderly individuals in order to more fully understand PCC in nursing homes.	Family members of elderly people with dementia (*n* = 9): Female (*n* = 7), male (*n* = 2), age 43–85 years (average 60.9). Relationships: spouses (*n* = 2) and daughters (*n* = 4), son (*n* = 1), and daughters‐in‐law (*n* = 2). 5 nursing homes in South Korea.	Semi‐structured in‐depth interviews (one‐on‐one) in a quiet place, 30‐90 minutes. Field notes and audio recording. The main questions: “What do you think should be included in person‐centred care in nursing homes?”, “Person‐centred care is necessary for the elderly with dementia in nursing homes. Why do you think it is necessary?”, “What do you think is necessary for person‐centred care in nursing homes?”.	The seven‐step phenomenological analysis method	Four theme clusters: The elderly at the centre of all decisions. Partnership between family and staff. Nursing home‐like home. Professional dementia care.
Kusmaul and Tucker (2020), USA	Qualitative (Descriptive qualitative study)	To understand how people in different positions experience PCC by exploring various stakeholders and comparing their perspectives.	Residents (*n* = 10), family members (*n* = 8), DCW from one continuing care retirement community (*n* = 9), managers (*n* = 5): females (*n* = 24), males (*n* = 8); age 20–99 years; White (*n* = 23), African American (*n* = 4), Hispanic (*n* = 2), Asian American (*n* = 2), and Central American (*n* = 1). 5 nursing homes in Baltimore, Maryland, USA.	Semi‐structured in‐depth interviews (face‐to‐face and one family member who participated via video call) in a quiet place, 14–56 min. The main questions on four practice areas: consistent assignment, meal choice, waking/bedtime, and bathing.	Content analysis	It is difficult to balance the resident's needs and safety. Leaders should provide education to residents, families, and staff. Consistent assignments contributed to the staff and resident collaboration. Meal choices: available all day, every day, and several places to eat. Waking: cultural change influenced waking practices. Bathing: flexible in the time and frequency.
Parkinson and Thompson (2022), UK	Qualitative (Descriptive qualitative study)	To explore care home staff's views/experiences of the prevalence of obesity in care homes, and challenges/facilitators associated with any rise in applications for placements.	Managers (*n* = 7), RNs (*n* = 9), RMNs (*n* = 2), Student nurses (*n* = 2), Older person specialist nurses (*n* = 2), healthcare assistants (*n* = 5), chefs (*n* = 6) 7 care homes in Northeast England.	Focus group interviews (2–8 participants) no longer than 1 h. Interview questions explored care home staff's views/experiences of older people with obesity (assessment for their admittance, care practices, challenges/facilitators, prevalence of obesity in care homes, policies and practice guidelines for managing older people with obesity, access to resources/levels of support from other health/social care professionals, recommendations for improving support).	Thematic analysis	Care home staff's ability to deliver person‐centred care. The importance of appropriate support/recognition of this as an emergent issue to be addressed at a higher executive level and by health/social care authorities. Three key types of resources/facilities of PCC: building design and environment, equipment and furniture, and staffing.
Vassbø et al. (2019), Norway, Australia, and Sweden	Phenomenological‐hermeneutical design.	To illuminate the meaning of working in a person‐centred way as experienced by nursing home staff, focusing on their satisfaction from work.	RNs (*n* = 10), ENs (*n* = 12), CAs (*n* = 2), personal carers (*n* = 2), occupational therapists (*n* = 2), physiotherapists (*n* = 1): females (*n* = 27), males (*n* = 3); age 22–65 years; experience working 1‐42 years. 3 nursing homes in a large city in Norway, and in rural areas in Australia and Sweden.	Individual interviews in the separate rooms in the nursing homes where participants could talk without being interrupted, 30–60 min. The main questions: ‘What do you see as the most important when working in a person‐centred way?’, What are your experiences?’, and ‘What do the experiences mean to you in your day‐to‐day work?’.	Lindseth and Nordberg's phenomenological‐hermeneutical method.	Two themes and six subthemes: Meeting individual residents' needs and preferences in close relationships: being in close family‐like relationships with residents, understanding residents’ rhythms and preferences as the basis of daily work plans, and doing the ‘little extra’ for residents. Meeting shared goals in collaborative teams: being part of a supportive team, working toward a collective practice, and sharing professional values.
Lynch et al. (2018), Ireland	Qualitative (Descriptive qualitative study, a complex action research study)	To implement and evaluate the effect of using the Person‐Centred Situational Leadership Framework to develop person‐centred care within nursing homes.	Residents (*n* = 8), RNs and carer (*n* = 6), leaders (*n* = 6) The private nursing home in the Republic of Ireland.	Observation of all team members using WCCAT to analyse the culture of the setting where the leader works. Critical and reflective dialogues to generate a deep understanding of the culture and the experiences of the leader. Narrative interviews (2 times) to gain an understanding of how residents perceived leadership. Focus groups with staff members and leaders, the questions focus on what they saw as the particular skills required by the leader to lead person‐centred care effectively and how they felt the PCSLF had or had not contributed to the developmental level of the team in the household. Reflective field notes in the whole process.	Thematic analysis and inductive approach.	Seven core attributes of the leader that facilitate person‐centredness: Relating to the essence of being. Harmonising actions with the vision. Balancing concern for compliance with concern for person‐centredness. Connecting with the other person in the instant. Intentionally enthusing the other person to act. Listening to the other person with the heart. Unifying through collaboration, appreciation, and trust.
Donnelly and MacEntee (2016), Canada	Qualitative (Descriptive qualitative study)	To describe and reflect the perceptions of residents about the care they received and their opinions on how organisational behaviour in the facilities influenced this care.	Older residents (*n* = 23): females (*n* = 16), males (*n* = 7); age 58–97 years. 7 long‐term care facilities purporting to provide person‐centred care.	Individual interviews in a quiet area, 30–120 min, and field notes. The main questions: “Tell me about some of the things you like to do” and “Tell me about the care you’ve received here”. 24‐hour observations to examine interactions between residents and staff, staff and management.	Framework analysis is an analysis influenced by Barber's theory of human relationships.	Three themes emerged: The caring environment The preservation of dignity The maintenance of personal autonomy.
Chenoweth et al. (2015), Australia	Qualitative (Descriptive qualitative study)	To understand the inconsistencies in PerCEN study findings for PCC and PCE and the factors that they believed both enabled and inhibited the implementation of PCC and PCE.	Care managers (*n* = 29), nurses and care staff (*n* = 70), family members (*n* = 73) Government‐accredited RACF in NSW, Australia.	Individual semi‐structured interviews with care managers, nurses, and care staff. Telephone surveys with family members. Survey and face‐to‐face interview questions: participant perceptions of changes in service quality and resident outcomes over the course of the study, and on enablers and barriers to PCC and/or PCE if introduced. 131 field note entries recorded by the person‐centred care and environment facilitators.	Content analysis	The person‐centred model: improved flexibility in care regimens, enhanced staff's attention to resident needs, reduced resident agitation, and improved their well‐being. Barriers and enablers: managerial leadership and support, staff capacity, effective communication and teamwork, care service flexibility, and staff education on how to focus care on the person's well‐being.
Røsvik et al. (2011), Norway	Qualitative (Descriptive qualitative study)	To conduct an initial evaluation of a model aimed at facilitating the application of the VIPS framework for PCC for persons with dementia.	Staff from NhA (*n* = 122) Staff from NhB (*n* = 110) 2 nursing homes in Norway	Focus groups (separate RNs and ANs in each NH). The main questions: how the model fitted with their form of organisation, experience of roles and functions, support needed and advice on alterations.	Content analysis	Four main themes: Legitimacy in the staff. Facilitation of the staff's use of knowledge about PCC. Support of the RP's facilitating role. The leading RN's authority in support of the legitimacy of the model.
Edvardsson et al. (2010), Australia	Qualitative (Descriptive qualitative study)	To describe the content of person‐centred care as described by people with dementia, family members, and staff in residential aged care.	Staff (*n* = 37), people with early onset dementia (*n* = 11), family members of patients with dementia (*n* = 19) Residential aged care in metropolitan and rural areas in Victoria, Australia.	The face‐to‐face interviews with family members and focus groups with staff and people with early onset dementia in a private room, 45 min–2 h. The interviews explored what the participants perceived to be person‐centred care, the meaning of high‐quality or low‐quality care in residential facilities; experiences of respite or residential care and on what grounds a decision was made about which aged care facility was chosen as a place where the person with dementia would live.	Content analysis	The core of person‐centred care is promoting a continuation of self and normality. Five content categories: knowing the person, welcoming family, providing meaningful activities, being in a personalised environment experiencing flexibility and continuity.

Abbreviations: ANs, assistant nurses; CAs, care assistants; DCW, direct care worker; Ens, enrolled nurses; NH, nursing home; NhA, nursing home A; NhB, nursing home B; PCE, person‐centred environment; PerCEN, the person‐centred dementia care and environment; PPC, person‐centred care; RACF, residential aged care facilities; RMNs, Mental health nurses; RNs, registered nurses; RP, resource person; VIPS, Valuing people, Individualised care, Personal perspectives, and Social environment.

### 3.2 Study Settings

Five studies were conducted in Australia (Roberts et al. 2015; Chenoweth et al. 2015; Edvardsson et al. 2010; Taylor et al. 2015; Toit et al. 2020), four in Sweden (Alexiou et al. 2021; Bökberg et al. 2019, 2020; Edvardsson et al. 2014), and Canada (Donnelly and MacEntee 2016; Hunter et al. 2016; Caspar et al. 2013; Williams et al. 2015), and three in the USA (Kusmaul and Tucker 2020; Coleman and Medvene 2013; Passalacqua and Harwood 2012). Two studies were conducted in each country of the United Kingdom (Parkinson and Thompson 2022; Ballard et al. 2018), Korea (Kim and You 2022; Choi et al. 2021), Norway (Røsvik et al. 2011; Jacobsen et al. 2017), and Portugal (Barbosa et al. 2017, 2015), one in Ireland (Lynch et al. 2018), Germany (Quasdorf et al. 2016), and Finland (Pakkonen et al. 2023). Three studies were conducted in multiple countries (Australia, Norway, and Sweden) (Vassbø et al. 2019; Sjögren et al. 2022; Lood et al. 2020).

### 3.3 Study Design

Quantitative studies (*n* = 12) used various designs including four experimental studies (Bökberg et al. 2019; Ballard et al. 2018; Barbosa et al. 2017; Passalacqua and Harwood 2012), three quasi‐experimental (Pakkonen et al. 2023; Edvardsson et al. 2014; Coleman and Medvene 2013), two multi‐centre, non‐equivalent controlled group before‐after designs (Sjögren et al. 2022; Lood et al. 2020), two correlational studies (Hunter et al. 2016; Caspar et al. 2013), and one controlled prospective cohort study (Alexiou et al. 2021). Qualitative studies (*n* = 9) employed a range of methods, including individual interviews, focus groups, and nonparticipatory observations. These studies utilised diverse designs, comprising two phenomenological approaches (Kim and You 2022; Vassbø et al. 2019) and seven descriptive qualitative designs (Chenoweth et al. 2015; Kusmaul and Tucker 2020; Parkinson and Thompson 2022; Lynch et al. 2018; Donnelly and MacEntee 2016; Røsvik et al. 2011; Edvardsson et al. 2010). Mixed method studies (*n* = 9) had convergent parallel (Bökberg et al. 2020; Quasdorf et al. 2016; Taylor et al. 2015), embedded (Toit et al. 2020), and sequential explanatory mixed methods designs (Roberts et al. 2015; Barbosa et al. 2015; Choi et al. 2021; Jacobsen et al. 2017; Williams et al. 2015).

### 3.4 Study Populations

The 30 included studies comprise 5049 participants. Of these, four collected data from older persons (*n* = 1206), two from family caregivers (*n* = 468), and 15 from healthcare providers (*n* = 2278). Three studies collected data from older persons, family caregivers, and healthcare providers (*n* = 37, 42, and 69, respectively). Five studies collected data from older persons and healthcare providers (*n* = 459 and 318, respectively). One study collected data from family caregivers and healthcare providers (*n* = 73 and 99, respectively). Two studies included older people with dementia (Edvardsson et al. 2010; Ballard et al. 2018).

### 3.5 Study Quality Appraisal

The assessment of 30 studies using the MMAT criteria indicated variability in quality, with an overall average rating classified as moderate. Several studies demonstrated strong methodological rigour, and a few showed significant areas for improvement, especially in meeting the quantitative and mixed methods criteria. The primary weaknesses identified were the lack of clarity in specifying the randomisation method, the absence of the double‐blind technique in randomised controlled trials, an unclear definition of inclusion and exclusion criteria, and failure to effectively integrate results in mixed method studies. MMAT assessments for all included studies are presented in Table 4.

**TABLE 4:** Study quality assessment of the included studies using the mixed method appraisal tool (MMAT), version 2018.

Citation	Screening questions		1. Qualitative					2. Quantitative, randomised controlled trials					3. Quantitative, non‐randomised					4. Quantitative descriptive					5. Mixed methods				
	Q1	Q2	1.1	1.2	1.3	1.4	1.5	2.1	2.2	2.3	2.4	2.5	3.1	3.2	3.3	3.4	3.5	4.1	4.2	4.3	4.4	4.5	5.1	5.2	5.3	5.4	5.5
Edvardsson et al. (2010)	✓	✓	✓	✓	✓	✓	✓																				
Røsvik et al. (2011)	✓	✓	✓	✓	✓	✓	✓																				
Chenoweth et al. (2015)	✓	✓	✓	✓	✓	✓	✓																				
Donnelly and MacEntee (2016)	✓	✓	✓	✓	✓	✓	✓																				
Lynch et al. (2018)	✓	✓	✓	✓	✓	✓	✓																				
Vassbø et al. (2019)	✓	✓	✓	✓	✓	✓	✓																				
Kusmaul and Tucker (2020)	✓	✓	✓	✓	✓	✓	✓																				
Kim and You (2022)	✓	✓	✓	✓	✓	✓	✓																				
Parkinson and Thompson (2022)	✓	✓	✓	✓	✓	✓	✓																				
Coleman and Medvene (2013)	✓	✓						✓	✓	✓	✓	✓															
Barbosa et al. (2017)	✓	✓						✓	✓	✓	✘	✓															
Ballard et al. (2018)	✓	✓						?	✓	✘	✓	✓															
Passalacqua and Harwood (2012)	✓	✓											?	✓	✓	✘	✓										
Caspar et al. (2013)	✓	✓											✓	✓	✓	✓	✓										
Edvardsson et al. (2014)	✓	✓											?	✓	✓	✘	✓										
Bökberg et al. (2019)	✓	✓											?	✓	✘	✘	✓										
Lood et al. (2020)	✓	✓											✓	✓	✓	✘	✓										
Alexiou et al. (2021)	✓	✓											✓	✓	✓	✘	✓										
Sjögren et al. (2022)	✓	✓											✓	✓	✘	✓	✓										
Pakkonen et al. (2023)	✓	✓											✓	✓	✘	✓	✓										
Hunter et al. (2016)	✓	✓																✓	✓	✓	✓	✓					
Barbosa et al. (2015)	✓	✓	✘	✓	✓	✓	✓	✓	✓	✘	✓	✓											✓	✓	✓	✓	✓
Roberts et al. (2015)	✓	✓	?	✓	✘	✓	✓						✓	✓	✓	✘	✓						✓	✘	✘	✓	✘
Taylor et al. (2015)	✓	✓	?	✓	✓	✓	✓	?	✓	✓	✘	✓											✓	✓	✓	✓	✓
Williams et al. (2015)	✓	✓	✓	✓	✓	✓	✓						✓	✓	✓	✓	✓						✓	✓	✓	✓	✓
Quasdorf et al. (2016)	✓	✓	?	✓	✓	✓	✓						?	✓	✘	✘	✘						✓	✓	✓	✓	✘
Jacobsen et al. (2017)	✓	✓	✓	✓	✓	✓	✓	✓	✓	✘	✓	✓											✓	✓	✓	✓	✓
Bökberg et al. (2020)	✓	✓	✓	✓	✓	✓	✓						✓	✓	✓	✘	✓						✓	✓	✓	✓	✓
Toit et al. (2020)	✓	✓	?	✓	✓	✓	✓						✓	✓	✓	✘	✓						✓	✓	✓	✓	✓
Choi et al. (2021)	✓	✓	?	✓	✓	✓	✓	?	?	✓	✘	✓											✓	✓	✓	✓	✘

*Note:* Yes = ✓ No = ✘ Can’t tell = ?. Q1 = Are there clear research questions?, Q2 = Do the collected data allow to address the research questions?, 1.1 = Is the qualitative approach appropriate to answer the research question?, 1.2 = Are the qualitative data collection methods adequate to address the research question?, 1.3 = Are the findings adequately derived from the data?, 1.4 = Is the interpretation of results sufficiently substantiated by data?, 1.5 = Is there coherence between qualitative data sources, collection, analysis and interpretation?, 2.1 = Is randomisation appropriately performed?, 2.2 = Are the groups comparable at baseline?, 2.3 = Are there complete outcome data?, 2.4 = Are outcome assessors blinded to the intervention provided?, 2.5 = Did the participants adhere to the assigned intervention?, 3.1 = Are the participants representative of the target population?, 3.2 = Are measurements appropriate regarding both the outcome and intervention (or exposure)?, 3.3 = Are there complete outcome data?, 3.4 = Are the confounders accounted for in the design and analysis?, 3.5 = During the study period, is the intervention administered (or exposure occurred) as intended?, 4.1 = Is the sampling strategy relevant to address the research question?, 4.2 = Is the sample representative of the target population?, 4.3 = Are the measurements appropriate?, 4.4 = Is the risk of nonresponse bias low?, 4.5 = Is the statistical analysis appropriate to answer the research question?, 5.1 = Is there an adequate rationale for using a mixed methods design to address the research question?, 5.2 = Are the different components of the study effectively integrated to answer the research question?, 5.3 = Are the outputs of the integration of qualitative and quantitative components adequately interpreted?, 5.4 = Are divergences and inconsistencies between quantitative and qualitative results adequately addressed?, 5.5 = Do the different components of the study adhere to the quality criteria of each tradition of the methods involved?

### 3.6 The Characteristics of the Care Models and Interventions

Care models or interventions to enhance person‐centred care in long‐term care facilities were mainly delivered through education programs for staff (Roberts et al. 2015; Pakkonen et al. 2023; Sjögren et al. 2022; Alexiou et al. 2021; Lood et al. 2020; Barbosa et al. 2017, 2015; Edvardsson et al. 2014; Coleman and Medvene 2013; Passalacqua and Harwood 2012; Bökberg et al. 2020; Jacobsen et al. 2017; Taylor et al. 2015; Toit et al. 2020; Williams et al. 2015). These programs varied in content, including concepts of person‐centredness, communication and relationship skills, leadership, dementia care, palliative care, organisational culture and tailored person‐centred activities. A peer‐enablement programme was also used to promote person‐centred care (Toit et al. 2020), while two studies focused on implementing and evaluating person‐centred care processes such as dementia care mapping (Choi et al. 2021; Quasdorf et al. 2016). Educational delivery methods commonly included lectures, workshops, group discussions, and reflective practice, with intervention durations ranging from 8 weeks to 24 months.

### 3.7 Integrated Findings

The effectiveness and experiences of various care models and interventions to improve person‐centred care have been widely studied. Seventeen studies have demonstrated positive effects of care models and interventions to improve person‐centred care for older people in long‐term care facilities (Roberts et al. 2015; Pakkonen et al. 2023; Sjögren et al. 2022; Lood et al. 2020; Bökberg et al. 2019, 2020; Ballard et al. 2018; Barbosa et al. 2017, 2015; Edvardsson et al. 2014; Coleman and Medvene 2013; Passalacqua and Harwood 2012; Choi et al. 2021; Jacobsen et al. 2017; Quasdorf et al. 2016; Taylor et al. 2015; Toit et al. 2020), while other studies have reported no significant effects on these groups (Alexiou et al. 2021; Williams et al. 2015). This review integrates findings on both the effectiveness and experiences associated with these care models and interventions, offering a comprehensive analysis of their impact. Five themes were generated.

#### 3.7.1 Theme 1: Personalised Preference, Social Engagement and Well‐Being

Six studies, both quantitative and qualitative, demonstrated that the interventions such as the Well‐being and Health for People with Dementia (WHELD) intervention, person‐centred interventions, and the ABLE model (Abilities and capabilities of the resident, Background of the resident, Leadership, education training and organisational culture change, and Physical environment) contributed to improving the quality of life and well‐being of residents, aligning with the fundamental objective of person‐centred care (Roberts et al. 2015; Chenoweth et al. 2015; Kim and You 2022; Edvardsson et al. 2010; Sjögren et al. 2022; Ballard et al. 2018). Residents in long‐term care facilities could directly benefit from physical, mental, and social support that enhanced their overall well‐being within their living environment.

Qualitative evidence illustrates the positive impact of person‐centred care, as one family caregiver described her mother's experience of increased autonomy and enjoyment through personalised activities:

She enjoys having her own space and making craft objects each day and engages in conversation with her new roommate. She is walking around the facility and the garden and so getting the physical activity she needs. She enjoys choosing meals and appears to be happy and relaxed. Her appetite has improved (Chenoweth et al. 2015).

Moreover, tailoring services to align with individual preferences, including attentiveness to dietary habits, enhanced residents' well‐being and overall experience. Qualitative findings illustrate this concept, as one family caregiver noted:

Well, it deals with the individual. For example, my mother is used to drinking lots of water not tea or coffee so it's about giving her what she wants. It's about attending to people's different food preferences and individuals' different ways of doing things (Edvardsson et al. 2010).

Other aspects of person‐centred care were also emphasised such as listening to older people and making sure their voices were heard and acted upon:

My mother's former habits didn't change even when I came to the nursing home. She had to brush her teeth immediately after eating and had to change her underwear every day. Even here, I know my mother's personality and recognise it fully (Kim and You 2022).

Furthermore, engaging in meaningful activities was reported to help foster a sense of self‐worth and enhance overall well‐being. Evidence demonstrated that allowing residents to perform tasks according to their abilities could maintain their self‐esteem and sense of independence. Even small decisions, such as choosing daily attire, contributed to their feeling of participation and control over their lives:

It is important to give mother the opportunity to perform tasks according to her ability, as if you don't use it, you lose it. It also gives her the opportunity to keep up her self‐esteem as she feels like she can still participate in her life. I think that it still is important that she makes some small decisions like what she might wear for the day, as it doesn't really matter what she decides (Edvardsson et al. 2010).

The findings highlighted the significance of providing residents with activity options and opportunities for involvement in their lives in long‐term care facilities.

These qualitative insights align with quantitative findings demonstrating the effectiveness of interventions in improving residents' quality of life. Specifically, the Well‐being and Health for People with Dementia (WHELD) intervention resulted in a significant enhancement in quality of life (mean difference 2.54, *p* = 0.0042) compared to standard care (Ballard et al. 2018). Additionally, other interventions designed to improve person‐centred care were associated with increased resident thriving, as evidenced by a mean difference of 4.2 between pre‐ and post‐intervention assessments (T0‐T1), with sustained but smaller improvements (mean difference = 0.5) in subsequent assessments (T1‐T2) (Sjögren et al. 2022). The quantitative data are presented in Table 5.

**TABLE 5:** Quantitative evidence on person‐centred care interventions.

Intervention/Study	Study design	Main outcomes	Effects
The Well‐being and Health for People with Dementia (WHELD) intervention (35)	RCT	Quality of life	Significant improvement compared with standard care (MD = 2.54, *p* = 0.004)
		Agitation	Reduced agitation (CMAI Z = 2.68, *p* = 0.008; d = 0.23)
		Neuropsychiatric symptoms	Reduced Neuropsychiatric symptoms (NPI‐NH Z = 3.52, *p* < 0.001; d = 0.30)
Person‐centred care interventions (31)	Pre–post	Resident thriving	Increased thriving post‐intervention (T0–T1: MD = 4.2); sustained but smaller improvement (T1–T2: MD = 0.5)
The ABLE model, a person‐centred care approach incorporating Montessori‐based activities for people with dementia (14)	Pre–post	Agitated behaviours	Significant reductions in physically non‐aggressive behaviours (*t* = 6.873, df = 14, *p* < 0.001) Significant reductions in verbally agitated behaviours (*t* = 6.632, df = 14, *p* < 0.001)
Educational programme (33)	Two‐group comparison	Relatives’ satisfaction	No significant group differences: safety (*β* = 0.67, *p* = 0.01) and hospitality (*β* = 0.32, *p* = 0.01) were significantly associated with satisfaction

In addition, both qualitative and quantitative findings indicate that person‐centred interventions contributed to reductions in agitation. Staff members reported that increased engagement efforts allowed residents to participate in meaningful and enjoyable activities (e.g., singing, dancing, games, etc.), leading to decreased agitation and an improved quality of life:

Staff have been putting in more effort to involve the residents … improved their quality of life as they actually get to enjoy their time at the facility, and they are less agitated because they get to engage in something meaningful and enjoyable (Chenoweth et al. 2015).

This is supported by quantitative data from the ABLE model, a person‐centred care approach incorporating Montessori‐based activities for people with dementia, which demonstrated significant reductions in physically non‐aggressive behaviours (*t* = 6.873, df = 14, *p* < 0.001) and verbally agitated behaviours (*t* = 6.632, df = 14, *p* < 0.001) (Roberts et al. 2015). Similarly, the WHELD intervention resulted in significant reductions in agitation (CMAI Z score 2.68, *p* = 0.0076; mean difference 4.27, SEM 1.59; 95% CI −7.39, −1.15; Cohen's D 0.23) and neuropsychiatric symptoms (NPI‐NH Z score 3.52, *p* < 0.001; mean difference 4.55, SEM 1.28; 95% CI −7.07, −2.02; Cohen's D 0.30) (Ballard et al. 2018). The quantitative data are presented in Table 5.

While tailoring care to residents' preferences is essential, staff shortages remain a significant challenge within the healthcare system, particularly in long‐term care facilities. Evidence has shown that these workforce constraints hinder the effective implementation of person‐centred care and compromise the overall quality of care provided in such settings. As one staff member shared:

I know the SPEC model (SPEC is the Systems for Person‐Centred Elder Care) is good, but we don't have human resources and time to use it. There is no time to feed the result of needs assessments and care plans into the SPEC model during working hours. If I want to use the SPEC model, I may have to work overtime (Choi et al. 2021).

Moreover, the workload prevented staff from having enough time to encourage resident participation in care. One staff member stated:

We would like to have more possibilities to let them [residents] participate, but we can't (Barbosa et al. 2015).

As a result, staff focused on routine tasks rather than person‐centred care. A resident shared their experience:

They have so much to do, it is rather difficult to reach them, they are not more than two at each ward, they are cleaning, making the beds. I mean they must do that—if it not that anyone is dying (Bökberg et al. 2020).

Additionally, staff shortages contributed to residents feeling unsafe, as illustrated by one resident's concern:

No one is coming in on the evening to say good night I can fall into the floor, I can even lie and die. How long shall I be laying before they discover when they don't come in on the evening (Bökberg et al. 2020)

#### 3.7.2 Theme 2: Autonomy and Dignity

Two studies, one quantitative and one qualitative, highlighted the importance of autonomy and dignity for residents in long‐term care facilities (Donnelly and MacEntee 2016; Hunter et al. 2016). Autonomy is a fundamental component of person‐centred care, yet it remains a significant challenge for staff in long‐term care facilities. The quantitative outcomes underscored the critical role of a person‐directed environment in preserving autonomy among older residents (*p* < 0.05). The presence of a person‐directed environment care model that prioritises personal preferences and individuality was positively associated with greater autonomy (Hunter et al. 2016). This statistical association is echoed in qualitative findings, where residents often described a loss of autonomy when care routines were imposed without regard for their personal needs. For instance, one resident expressed frustration with a physiotherapist who insisted they would need to walk more:

I get dizzy and my legs start shaking, and I don't want to fall down and break something. So, I've had an argument with our physiotherapist [who said] ‘you should walk, you should walk more’. Well … they're my legs, and I know if I can stand up or not, and I told her ‘get off my back,’ so she left me alone (Donnelly and MacEntee 2016).

Dignity is another essential aspect of person‐centred care, which has been frequently overlooked in routine care practices. A resident shared their experience of feeling terrible:

I've always got pads on. To begin with, I thought it was absolutely foul. In the hospital, they gave you the bedpan, but they don't give you that here. They don't approve of that, so what can I do? You lie in your own filth, but then they come and take the dirty pad off, put a clean one on, and away they go again. The routine is exactly the same for everyone (Donnelly and MacEntee 2016).

These narratives illustrate the challenges long‐term care facilities face in balancing efficiency with individualised, dignified care. Several factors have contributed to this issue, including resource limitations (understaffing and time constraints), lack of staff training on autonomy and dignity, and an institutional culture that prioritises efficiency over personalised care (Donnelly and MacEntee 2016).

#### 3.7.3 Theme 3: A Home‐Like Environment

Two studies identified the creation of a home‐like environment as a key factor in enhancing the well‐being of residents in long‐term care facilities (Kim and You 2022; Edvardsson et al. 2010). Building an environment in a nursing home that looks like an older person's home is important for an older person in a nursing home because it helps them become familiar with the environment:

Being homely means being able to look out the window and liking what you're looking at and inside you can have pictures on the wall and you can have your own bathroom and it feels like it is your own bedroom. Just normal things around you like a garden and animals, that's what I see as homely (Edvardsson et al. 2010).

Providing care for older people like family enhanced both their well‐being and satisfaction while also fostering trust and fulfilment among their relatives:

It seems like the caregivers think of the elderly as their family. I think they take care of them like their own parents. They are so nice to my mom. The staff are so playful, and they love the residents. That's why they are so reliable and trustworthy (Kim and You 2022).

Furthermore, a peaceful and welcoming environment could foster a sense of safety and comfort for people with dementia while also enhancing family members satisfaction:

My mom has dementia and obsessive‐compulsive symptoms. It's hard when the stuff my mom's obsessed with disappears. The nursing staff realized this and asked me to bring my mom's favourite things. My mom's comfortable right now, so she talks less about going home (Kim and You 2022).

These findings highlight the importance of establishing a home‐like environment in nursing homes to ensure that older people feel comfortable and familiar with their surroundings.

#### 3.7.4 Theme 4: Family Involvement and Satisfaction

Family caregivers expressed satisfaction with the care provided, particularly when they feel welcomed to participate in the care process by staff. Family caregivers actively collaborated with staff to ensure their relatives would receive the best care. They visited nursing homes freely and maintained open, ongoing communication with staff. As one family caregiver shared:

The nurses here are taking good care of my mom, but I've been taking care of her for a long time. If I suddenly notice my mom is getting sick, I'll tell the nurses in advance to get nutritional supplements. The nurses understand and appreciate our opinions (Kim and You 2022).

This highlights the mutual exchange of information between families and staff, reinforcing a collaborative caregiving approach.

These qualitative insights are supported by quantitative data from a two‐group comparison study evaluating the effectiveness of an educational programme focused on person‐centred care (a 14‐month education on person‐centredness, person‐centred care, thriving and caring environment for staff in the intervention group, and a one‐hour lecture before the intervention period for staff in the control group). The findings revealed that relatives of residents in both the intervention and control groups were satisfied with the quality of care. No statistically significant differences in satisfaction with care quality were found between the two groups. However, safety was significantly associated with relatives' overall satisfaction regarding the quality of care (*β* = 0.67, *p* = 0.01), while hospitality also demonstrated a strong, statistically significant relationship with satisfaction (*β* = 0.32, *p* = 0.01) (Lood et al. 2020). The quantitative data are presented in Table 5.

Both qualitative and quantitative data from included studies support the concept of promoting person‐centred care through family involvement and effective communication could enhance the satisfaction of both family members and staff in long‐term care facilities.

#### 3.7.5 Theme 5: Organisational and Staff Support

Organisational support is a key component in enhancing person‐centred care within long‐term care facilities, encompassing adequate staffing, effective leadership and a supportive organisational culture. Sufficient staffing is crucial for fostering effective teamwork in the care of older persons in long‐term care facilities:

My husband is a big man, so it's hard for one person to move him. Compared to the nursing home before, this place has more staff who help each other a lot. That's why I'm relieved (Kim and You 2022).

Another factor that contributed to the staff and residents' collaboration was consistent assignments for staff and continuity of care:

I've gotten to like and admire one person. I almost demand that she come every night though she's not here every night, so I can't (Kusmaul and Tucker 2020).

However, most staff focused on the daily routines of care more than taking time to talk to older people or creating suitable activities for them.

…they are doing their best, they need to share the food to everybody before they can sit down and talk to us. It must be stressful for them as well… there need to be more staff… but I am not complaining (Bökberg et al. 2020).

Additionally, leadership and organisational culture among staff are critical factors that could either support or hinder the adoption of person‐centred care. Effective leadership attracted skilled, motivated, and supportive nurses and care staff who were willing to learn. As a result, improvements in care quality and resident well‐being were noted:

The facility's reputation and public relations improve, with positive feedback from relatives and more new residents requesting entry (Chenoweth et al. 2015).

These findings indicated that an organisational support enabled trust, strong relationships, and high‐quality person‐centred care in long‐term care facilities.

In addition, collaboration and support among team members are essential components of effective teamwork. In particular, sharing professional expertise within the team facilitated collective efforts toward achieving shared goals:

I have experienced that colleagues that are sharing the goals and want to work in this field increase their professional competence and are more likely to wish to learn more, such as about environmental measures to promote person‐centred care, for instance (Vassbø et al. 2019).

Fostering effective communication within the team helped build a shared understanding of an older person and ensured care was tailored to meet the needs of older person:

At the case conference, care teams gathered and communicated about the patient, making it easier to understand the patient and get help from other teams to implement interventions for patient (Choi et al. 2021).

Furthermore, collaboration and mutual support through peer interaction could help address the unmet needs of older people:

everyone absolutely love it, love the problem‐solving, every single team, one team had chaos break out on a shift and the staff said could they meet without the team leaders—they just want to carry on with the problem‐solving (Toit et al. 2020).

A study implementing the Peer Enablement Programme found that 90.6% of nurses improved their ability to differentiate between delirium and dementia, while 87.8% demonstrated enhanced skills in observing and assessing pain in individuals with dementia (Toit et al. 2020). Another study showed that staff increased awareness of person‐centred care practices (Jacobsen et al. 2017).

## 4 Discussion

Following a systematic search of eight databases, 30 studies met the inclusion criteria for this review, reporting on the effectiveness and experiences of care models or interventions aimed to enhance person‐centred care for older persons in long‐term care facilities. These studies revealed that care models or interventions could enhance the quality of life and well‐being of residents while also reducing neuropsychiatric symptoms (Roberts et al. 2015; Chenoweth et al. 2015; Sjögren et al. 2022; Ballard et al. 2018). They also contributed to promoting person‐centred care by providing care based on residents' backgrounds, needs, preferences, and social interactions (Kim and You 2022; Edvardsson et al. 2010; Alexiou et al. 2021; Bökberg et al. 2019). Additionally, these care models supported the maintenance of residents' autonomy and dignity (Donnelly and MacEntee 2016; Hunter et al. 2016) and emphasised the creation of an environment tailored to their needs and familiarity (Chenoweth et al. 2015; Kim and You 2022; Edvardsson et al. 2010). From a family perspective, these care models encouraged family caregivers to be actively involved in care, which in turn increased their satisfaction with the care provided (Kim and You 2022; Lood et al. 2020). Implementing care models or interventions to enhance person‐centred care presents significant challenges for long‐term care facilities. Evidence shows that enhancing organisational support (Chenoweth et al. 2015; Kim and You 2022; Kusmaul and Tucker 2020; Bökberg et al. 2020), teamwork, and peer enablement (Roberts et al. 2015; Vassbø et al. 2019; Choi et al. 2021; Jacobsen et al. 2017; Taylor et al. 2015; Toit et al. 2020) is important to ensure the effective implementation of person‐centred care. The positive attitudes of involved individuals, well planned interventions, recruitment of champions who advocate for the care model, and involvement of qualified and experienced project coordinators also played crucial roles in ensuring effective implementation (Quasdorf et al. 2016). However, the influential factors such as staff shortage and workload were identified to hinder its implementation (Barbosa et al. 2015; Bökberg et al. 2020; Choi et al. 2021).

The effectiveness of these care models and interventions is not conclusive depending on their design and delivery. Some studies reported improvements in person‐centred climate scores, a validated tool that assesses how well a care environment supports person‐centred care by measuring aspects such as safety, everydayness and hospitality among both staff and residents. However, other studies found no significant effects (Alexiou et al. 2021; Caspar et al. 2013). The person‐centred climate scores were completed by both staff and residents. This discrepancy may be attributed to differences in intervention content, duration, and study methods and methodology, such as the inclusion of a control group versus a one‐group pre‐post design. Additionally, variations in participants' backgrounds, including cognitive and functional status, may have influenced outcomes, highlighting the need for further research to identify the most effective strategies for personalisation.

Previous studies have highlighted that person‐centred care is positively associated with the quality of life of older people in long‐term care facilities. It is built around key elements such as autonomy, dignity, individual needs, and the social interaction and environment, which lead residents to feel empowered, valued, involved, and connected through their care experience (Skoldunger et al. 2020; Kabadayi et al. 2020). The findings from this review are consistent with those studies, showing that the well‐being of older people improves when the services they receive meet their needs. The relevant findings from the included studies mainly indicated that understanding residents' backgrounds, personal preferences, social interactions, autonomy, and dignity enhances their well‐being and facilitates their adaptation to long‐term care environments (Roberts et al. 2015; Chenoweth et al. 2015; Kim and You 2022; Edvardsson et al. 2010; Sjögren et al. 2022; Ballard et al. 2018). However, this review revealed differing perspectives on autonomy and dignity. Although autonomy is widely acknowledged as a key aspect of person‐centred care, the findings indicate that preserving residents' autonomy continues to be a significant challenge in long‐term care settings. Some residents reported experiencing a loss of control in their daily lives, particularly when staff prioritised operational efficiency over individual preferences. These findings are consistent with previous studies, which suggest that autonomy can be compromised when staff rigidly follow routines, deliver care without considering residents' choices, or delay responding to their requests for assistance (Loon et al. 2021). Preserving the autonomy and dignity of older people remains a key challenge for long‐term care policy within residential care facilities.

For older people, this review indicated that creating an environment that reflects their personal histories and fosters a sense of familiarity can help them feel more comfortable in a new place. The prior research demonstrated that environmental modifications, such as private rooms, well‐defined spatial layouts, and offerings like homemade meals and rehabilitation services, contribute to creating a home‐like atmosphere. These factors have been shown to positively influence residents' moods and behaviours, enhancing their overall well‐being and comfort in long‐term care facilities (Bae and Kim 2024).

Beyond the physical environment, a culture of familial care and social interaction played a crucial role in enhancing residents' experiences. Family caregivers and staff who treated residents as valued individuals rather than passive care receivers contributed to a more positive and trusting atmosphere. This finding aligns with previous research indicating that most residents expressed satisfaction with the attentive care they received from staff, especially during times of illness or discomfort, as it helped create a homely and reassuring environment. Furthermore, residents valued the opportunity to spend quality time with their family members within the facility, reinforcing their sense of connection and emotional well‐being (Bae and Kim 2024). These findings suggest that fostering a home‐like setting in long‐term care facilities may enhance both physical and emotional comfort for residents, particularly those with dementia.

In addition, the review highlights the significance of family involvement, effective communication, and a supportive environment in enhancing family caregivers' satisfaction. Visiting their relatives freely and engaging in open communication fosters trust and collaboration (Kim and You 2022). These are consistent with findings from other research evidence, demonstrating that effective communication is an essential prerequisite for establishing trust and significantly influences the perceived quality of family caregivers' involvement in their relatives' daily lives. Family caregivers regarded informal interactions with staff as a form of effective communication, facilitating the development of personal connections between them and nursing home personnel. Consequently, interactions between family caregivers and staff can help strengthen trust and improve family caregivers' satisfaction with the care that their relative received in long‐term care facilities (Hoek et al. 2021).

In terms of organisational support, staffing levels emerged as a pivotal factor in the successful implementation of person‐centred care within long‐term care facilities. Existing evidence consistently demonstrates that sufficient staffing not only enables the development of effective teamwork but also ensures that residents receive timely and appropriate support (Baek et al. 2023). When combined with strong leadership and supervisor role modelling, adequate staffing contributes to the creation of family‐like bonds between staff and residents, fostering a care environment that is both personalised and high in quality (Spilsbury et al. 2024). Notably, adaptive leadership principles have been shown to enhance person‐centred care delivery at both the frontline and organisational levels (Kuluski and Reid 2021), reinforcing the importance of leadership in shaping care culture.

Despite these positive associations, staff shortages remain a critical barrier. Facilities with inadequate staffing often struggle to meet residents' needs and maintain safe care practices, leading to increased workloads, diminished staff well‐being, and compromised care quality (Barbosa et al. 2015; Bökberg et al. 2020; Choi et al. 2021; Spilsbury et al. 2024). In contrast, workforce stability supports continuity of care, allowing staff to build deeper relationships with residents and tailor care to individual preferences. This consistency not only improves care outcomes but also strengthens trust and emotional connection between caregivers and residents. The findings underscore that staffing adequacy and leadership are interdependent components essential to embedding person‐centred care in long‐term care settings. Addressing workforce challenges and promoting stable, well‐supported teams should be prioritised in future policy and practice to ensure sustainable improvements in care quality.

### 4.1 Implications for Practice and Future Research

The review presents valuable insight and a comprehensive understanding of the effectiveness and experiences of care models and interventions to improve person‐centred care. These findings have the potential to significantly impact clinical practice, inform policy and guideline development, enhance practice, and improve the effectiveness of person‐centred care in long‐term care facilities, thereby substantially influencing the care of older people. By acknowledging and addressing barriers while leveraging facilitators, organisations play a crucial role in providing more empathetic and tailored support to healthcare providers in long‐term care facilities.

Future work is required to evaluate the long‐term impact of person‐centred care for this population and identify effective strategies to strengthen organisational support, including leadership development, staff retention, and resource allocation, and evaluate how organisational culture influences the adoption and success of person‐centred care practices. Additionally, further efforts should focus on developing care models and interventions to address the needs of this population and evaluating their long‐term impact, particularly in low‐ and middle‐income countries.

### 4.2 Strengths and Limitations

This review includes studies published in English and Thai between 2000 and 2024, identified through searches of multiple databases, focusing on research that explores both historical and current care models or interventions aimed at improving person‐centred care. Systematic and rigorous methodological approaches to searching and data analysis have allowed for the creation of a high‐quality synthesis.

Inevitably, any review has some limitations. Although the search covered eight electronic databases, only studies published in English and Thai peer‐reviewed studies were included, so relevant evidence from other countries may have been missed which could shed light on this topic. Secondary research, editorials, expert opinions, conference proceedings, and grey literature were not considered in the search, potentially leading to the omission of relevant research.

## 5 Conclusion

This review highlighted the significance of tailored care, strong relationships, and supportive environments. The interventions effectively improved residents' quality of life and well‐being. It also reduced neuropsychiatric symptoms and promoted person‐centred care by considering residents' backgrounds, needs, activity preferences, and social interactions. These efforts also created an environment aligned with residents' needs and sense of familiarity, as reflected in the positive perceptions of thriving and satisfaction reported by both residents and their relatives. However, challenges in preserving autonomy and ensuring dignity underscore the need for resource allocation and staff training to address systemic barriers. Moreover, staff shortages and lack of managerial support may hinder successful implementation.

## Author Contributions

All authors have contributed to the conception and writing of the paper. The review protocol was written by K.U. and received approval from the supervisors, P.G. and N.E. K.U. was responsible for developing the search strategies, conducting all database searches, initially screening the literature, performing data analysis and interpretation, and drafting the manuscript. The second author (P.G.) reviewed the full text of the included papers and screened them for meeting inclusion or exclusion criteria. A third reviewer (N.E.) was available to provide an independent opinion on any dispute decisions. All authors have independently screened and collaboratively have been involved in the analysis and interpretation of data, critically appraising the content, revising the manuscript and approving the final version for publication.

## Funding

This research did not receive any specific funding from public, commercial or nonprofit organisations. However, the first author was awarded an academic scholarship to support her PhD programme from the Faculty of Nursing, Chiang Mai University, Chiang Mai, Thailand.

## Conflicts of Interest

The authors declare no conflicts of interest.

## Data Availability

The data that support the findings of this study are available on request from the corresponding author. The data are not publicly available due to privacy or ethical restrictions.
